# Extension of Dupilumab Injection Intervals in Chronic Rhinosinusitis with Nasal Polyps: A Real-World Study

**DOI:** 10.3390/ph19060961

**Published:** 2026-06-22

**Authors:** Michael Habenbacher, Ulrich Moser, Angelika Lang, Ahmed Abaira, Jakob Pock, Thomas Lampl, Alexandros Andrianakis

**Affiliations:** Department of Otorhinolaryngology, Medical University of Graz, 8010 Graz, Austria

**Keywords:** CRS, CRSwNP, biologics, dupilumab, real-life, dose tapering

## Abstract

**Background/Objectives**: Dupilumab is an effective long-term treatment for chronic rhinosinusitis with nasal polyps (CRSwNP), but continuous biweekly (Q2W) treatment is associated with high costs, cumulative drug exposure, and treatment burden. Extending injection intervals may reduce these burdens. This real-world study aimed to evaluate the feasibility and clinical outcomes of dupilumab interval extension compared with continued standard Q2W dosing. **Methods**: In this retrospective single-center study, 35 adults with CRSwNP who had received dupilumab 300 mg Q2W for >12 months underwent a stepwise interval-extension attempt (two-week increments, ≥6 months between steps) and were compared with 30 patients who continued Q2W dosing. Clinical outcomes were assessed every six months using the Sino-Nasal Outcome Test-22 (SNOT-22) and Nasal Polyp Score (NPS). **Results**: Of the 35 patients, 19 patients (54%) reached a dosing interval of Q4W or longer, and eight patients (23%) maintained a partial extension to Q3W after not tolerating Q4W. The remaining eight patients (23%) returned to Q2W. Thus, 27 patients (77%) stayed on an extended interval beyond the standard Q2W regimen, which was maintained throughout follow-up without reversion to a shorter regimen. SNOT-22 and NPS improved significantly after dupilumab initiation and remained stable throughout follow-up in both groups, with no significant between-group differences in longitudinal outcomes. No patient needed rescue treatments during follow-up. **Conclusions**: In selected clinically stable patients with CRSwNP, dupilumab interval extension appeared feasible and was associated with maintained disease. Individualized dose tapering, including intermediate intervals such as Q3W, may reduce treatment burden without compromising disease control. Larger prospective studies are needed to define optimal extension protocols and identify predictors of successful interval extension.

## 1. Introduction

Chronic rhinosinusitis with nasal polyps (CRSwNP) is a common chronic inflammatory disease of the nose and paranasal sinuses, affecting approximately 2–4% of the population in Western countries [1,2,3]. It is characterized by persistent sinonasal symptoms lasting for more than 12 weeks, including nasal obstruction or congestion, impaired sense of smell, rhinorrhea, post-nasal drip, and facial pain or pressure, together with the presence of inflammatory nasal polyps arising from the sinonasal mucosa [1]. Beyond its local inflammatory manifestations, CRSwNP is frequently associated with comorbid conditions including bronchial asthma and NSAID-exacerbated respiratory disease (N-ERD) [4]. Due to its chronic and recurrent course, CRSwNP imposes a substantial burden on quality of life and contributes to relevant healthcare costs [1,5,6].

In recent years, CRS has increasingly been understood as a heterogeneous disease that can be classified according to the predominant inflammatory pathways involved. This endotype-based approach distinguishes between type 1, type 2, and type 3 inflammation, with the differentiation between type 2 and non-type 2 disease being particularly important in clinical practice [7]. In Western populations, most patients with CRSwNP (approximately 85%) exhibit a type 2 inflammatory profile, characterized by eosinophilic inflammation and increased activity of the key-cytokines IL-4, IL-5, and IL-13. In contrast, type 1 inflammation is mainly associated with IFN-γ and IL-12, whereas type 3 inflammation is characterized by IL-17A and IL-22 [7,8,9]. These inflammatory processes are accompanied by epithelial barrier dysfunction, impaired mucociliary clearance, abnormal immune responses, and tissue remodeling. Although the exact etiological triggers of the inflammation remain incompletely understood, both host-related factors and environmental exposures, such as pathogens, allergens, and pollutants, are believed to contribute to disease development and persistence [1,10].

Standard treatment of CRSwNP includes long-term topical corticosteroids, short-term courses of systemic corticosteroids (SCS), and endoscopic sinus surgery (ESS) [1]. Despite these treatment options, many CRSwNP patients still suffer from persistent symptoms and nasal polyp recurrence [11,12,13]. A better understanding of the inflammatory mechanisms underlying CRSwNP has substantially changed the therapeutic landscape. In particular, the identification of type 2 inflammation as a key disease-driving pathway has enabled the development of targeted biologic treatments. Among these, monoclonal antibodies inhibit specific key cytokines or receptors that contribute to type 2 inflammation [14].

Dupilumab is a fully human monoclonal antibody that targets the IL-4 receptor α-subunit, thereby inhibiting both IL-4 and IL-13 signaling and reducing type 2 inflammation [15]. Its efficacy in CRSwNP has been demonstrated in randomized clinical trials and supported by several real-world studies [16,17,18,19]. As CRSwNP is a chronic disease, dupilumab is generally considered a long-term treatment option. Although dupilumab therapy at the approved dose of 300 mg every two weeks (Q2W) is highly effective, continuous treatment over years comes with practical challenges, including high cumulative drug exposure, treatment burden and rising healthcare costs [20,21]. In this context, dose-optimization strategies have gained increasing interest in the long-term management of biologic therapies. Extending dupilumab injection intervals may represent a practical approach to reduce treatment frequency, drug exposure, and healthcare costs while maintaining disease control [22]. Emerging real-world evidence suggests that extended dupilumab injection intervals, such as every four weeks (Q4W), may maintain clinical benefit without compromising efficacy. However, evidence supporting interval extension in CRSwNP remains limited [23,24,25,26,27,28,29]. Therefore, additional real-world data are urgently needed to better define the feasibility and effectiveness of extended dupilumab injection intervals.

At our department, dupilumab interval extension has been incorporated into routine clinical practice, providing an opportunity to evaluate this treatment strategy in a real-world setting. Therefore, this real-world study aimed to evaluate the feasibility and clinical outcomes of dupilumab interval extension in patients with CRSwNP.

## 2. Results

### 2.1. Patient Flow and Baseline Characteristics

**Figure 1:**
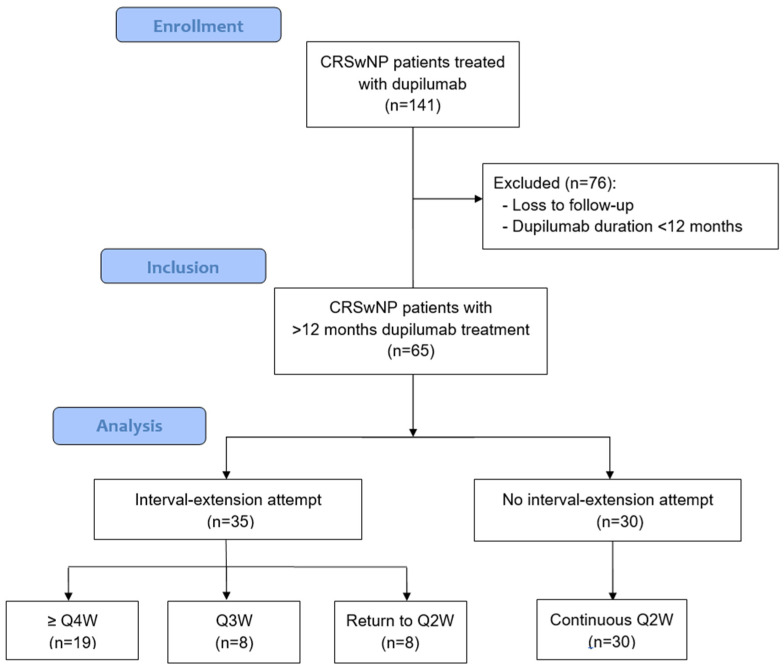
Patient flow chart.

A total of 141 patients with CRSwNP were treated with dupilumab at our center. Of these, 76 patients were excluded because they had a dupilumab treatment duration of ≤12 months or were lost to follow-up before reaching 12 months. The remaining 65 patients had received >12 months of dupilumab treatment. Among these patients, 35 had an interval-extension attempt, whereas 30 patients remained on continuous Q2W treatment without an interval-extension attempt. Patient flow is shown in Figure 1.

Demographic and baseline clinical characteristics stratified by interval-extension attempt status are presented in Table 1. Patients without a documented interval-extension attempt more frequently had coexisting asthma than patients with an interval-extension attempt (83% vs. 49%; *p* = 0.005). No significant differences were observed for the remaining baseline characteristics (all *p* > 0.05).

### 2.2. Treatment Outcomes of Patients with Interval-Extension Attempt

In patients who underwent an interval-extension attempt, SNOT-22 scores changed significantly over time (*p* < 0.001). The observed mean SNOT-22 score decreased from 48.4 ± 20.0 at baseline to 25.0 ± 18.24 after 6 months and remained substantially reduced throughout follow-up. Detailed values are summarized in Table 2. Pairwise comparisons showed significantly lower SNOT-22 scores at each follow-up visit compared with baseline after Bonferroni correction (all *p* < 0.001).

NPS also changed significantly over time (*p* < 0.001). Median NPS decreased from 5 (IQR, 4–6) at baseline to 1 (IQR, 0–2) after 6 months and remained low throughout the follow-up period. Pairwise comparisons against baseline showed significantly lower NPS values at all follow-up visits after Bonferroni correction (all *p* < 0.001). Detailed NPS values are presented in Table 3. Furthermore, none of the 35 patients required rescue SCS or salvage ESS during the entire follow-up period.

Mean blood EOS increased from 0.48 ± 0.34 × 10^9^/L at baseline to a peak of 0.63 ± 0.51 × 10^9^/L at 6 months; then gradually declined to 0.59 ± 0.48 × 10^9^/L at 12 months, 0.54 ± 0.43 × 10^9^/L at 18 months, and 0.54 ± 0.42 × 10^9^/L at 24 months; and remained below 0.5 × 10^9^/L throughout the remaining follow-up up to 60 months. Three patients (9%) developed dupilumab-induced blood hypereosinophilia (≥1.5 × 10^9^/L) after 6 months. In two of these patients, hypereosinophilia had resolved by the 12-month visit, while the third patient showed persistent values between 1.5 and 3.0 × 10^9^/L until the last available follow-up at 30 months without any associated clinical symptoms.

### 2.3. Outcome of Dupilumab Interval Extension

Of the 35 patients who underwent an extension attempt, 19 (54%) maintained an extension of ≥Q4W—with Q4W the most common interval and a small subset reaching Q6W to Q10W—while eight (23%) maintained a partial extension to Q3W after not tolerating Q4W, and eight (23%) reverted to Q2W. In these patients, inability to tolerate Q4W was based on patient-perceived recurrence or worsening of CRS-related symptoms after interval extension. Taken together, 27 patients (77%) achieved an extended interval (Q3W or longer) beyond the standard Q2W regimen. Detailed interval-extension findings are shown in Table 4.

**Figure 2:**
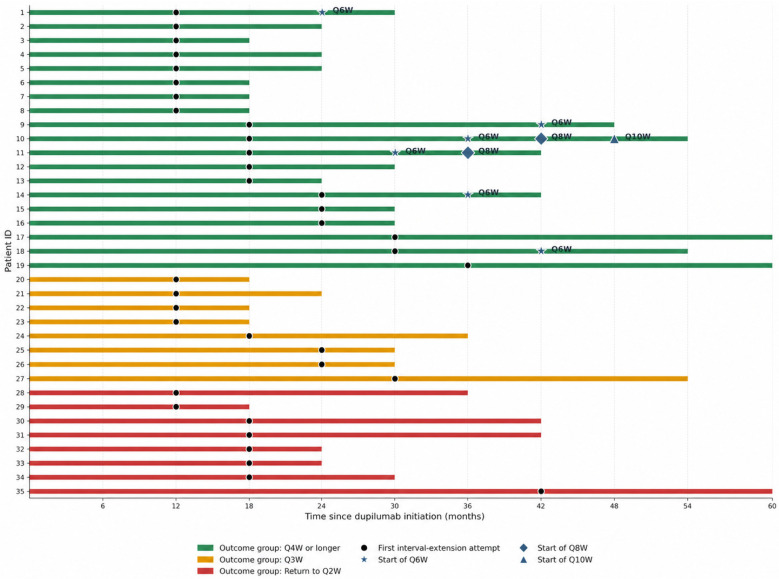
Swimmer plot of dupilumab extension trajectories.

None of the 27 patients who maintained an extended interval (≥Q3W) required a return to a shorter dosing interval during the entire follow-up period. The attempt of first interval extension was made after a median treatment duration of 18 months (range, 12–42 months). Specifically, interval extension was initiated after 12 months in 14 patients (40%), after 18 months in 10 (29%), after 24 months in six (17%), after 30 months in three (8%), after 36 months in one (3%), and after 42 months in one patient (3%). Individual patient-level trajectories, including follow-up duration, timing of the first interval-extension attempt, and achieved interval-extension outcome, are shown in Figure 2.

### 2.4. Clinical Outcomes During Interval Extension

The clinical course of patients with an extended dosing interval (n = 27) was assessed by comparing SNOT-22 and NPS values at three key time points: baseline (at biologic initiation), pre-extension (defined as the last visit immediately before the first attempt of interval extension), and post-extension (defined as the visit at the maximum reached injection interval, with 6 months on this interval).

**Figure 3:**
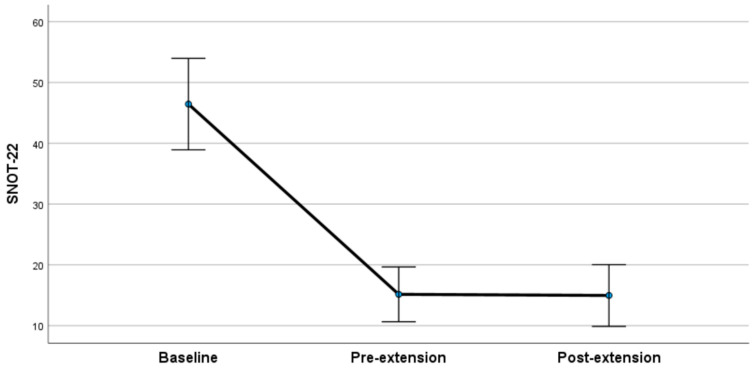
SNOT-22 scores at baseline, before and after dupilumab interval extension.

For SNOT-22, rmANOVA revealed a highly significant main effect (*p* < 0.001). Bonferroni-corrected pairwise comparisons demonstrated a significant reduction in SNOT-22 from baseline (46.4 ± 19.0) to pre-extension (15.1 ± 11.4; mean difference: −31.3, 95% CI −39.6 to −23.0; *p* < 0.001). Importantly, SNOT-22 values remained unchanged between pre-extension and post-extension (14.9 ± 12.8; mean difference: −0.2, 95% CI −3.4 to 3.7; *p* = 1.000). The reduction from baseline to the post-extension visit was likewise highly significant (mean difference: −31.5, 95% CI −40.1 to −22.8; *p* < 0.001). A clinically relevant worsening of SNOT-22 (increase ≥ 12 points [30]) between pre-extension and post-extension was observed in only one patient, whose post-extension score still remained below 40 points. The course of SNOT-22 before and during interval extension is illustrated in Figure 3.

**Figure 4:**
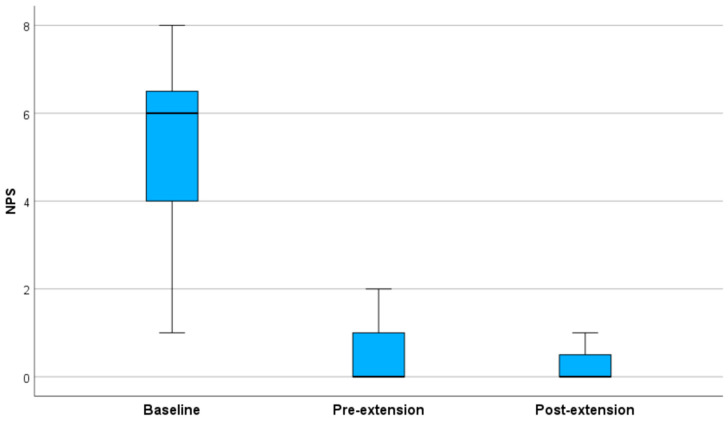
NPS at baseline, before and after dupilumab interval extension.

A similar pattern was observed for NPS. Median NPS decreased from 6 (IQR 4–7) at baseline to 0 (IQR 0–1) at pre-extension and remained at 0 (IQR 0–1) at post-extension. The Friedman test demonstrated a significant overall effect (*p* < 0.001). Bonferroni-corrected pairwise comparisons showed a significant reduction from baseline to pre-extension (*p* < 0.001) and from baseline to post-extension (*p* < 0.001) but no significant change between pre-extension and post-extension (*p* = 1.000). The NPS remained stable in all 27 patients. The NPS course before and during interval extension is shown in Figure 4.

### 2.5. Baseline Predictors of Interval Extension Outcome

To identify baseline factors associated with achieved interval extension, patients who reached ≥Q4W (n = 19) were compared with those who did not tolerate Q4W (<Q4W; n = 16), including patients maintained on Q3W (n = 8) and those who returned to Q2W (n = 8). No statistically significant differences were observed between the ≥Q4W and <Q4W groups for any assessed baseline characteristic (all *p* > 0.05; Table 5). In addition, an exploratory three interval-extension group analysis (≥Q4W, Q3W, return to Q2W) likewise did not identify significant differences across groups (all *p* > 0.05). Detailed results are presented in Table 5.

### 2.6. Comparison with Continuous Q2W Treatment

Out of the 65 patients who had received >12 months of dupilumab treatment, 30 patients remained on continuous Q2W treatment without an interval-extension attempt. Among these, six patients still reported subjective bothersome symptoms, while the remaining 24 patients either refused an interval extension attempt or were not offered at the discretion of the treating physician.

In continuous Q2W patients without an interval-extension attempt, SNOT-22 scores also changed significantly over time (*p* < 0.001). The mean SNOT-22 score decreased significantly from 57.9 ± 21.9 at baseline to 24.7 ± 19.8 after 6 months and stayed reduced throughout follow-up (all pairwise comparisons to baseline *p* < 0.05). NPS showed a similar longitudinal pattern, decreasing significantly from a median of 5 (IQR, 4–6) at baseline to 1 (IQR, 0–2) after 6 months and remaining low during follow-up (all pairwise comparisons to baseline *p* < 0.05). None of the 30 continuous Q2W patients required rescue SCS or salvage ESS during the entire follow-up period. Detailed results are shown in Tables S1 and S2.

**Figure 5:**
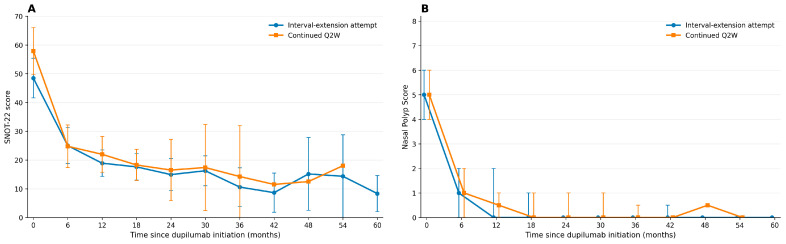
Longitudinal clinical outcomes by interval-extension attempt status. Panel (**A**) SNOT-22. Panel (**B**) NPS.

In the between-group comparison, no significant difference in the longitudinal SNOT-22 course was observed between patients with an interval-extension attempt and those continuing Q2W treatment without an attempt (group-by-visit interaction, *p* = 0.330). Similarly, the longitudinal NPS course did not significantly differ between groups (group-by-visit interaction, *p* = 0.782). The longitudinal courses of SNOT-22 and NPS in both groups are illustrated in Figure 5.

## 3. Discussion

This real-world study evaluated the feasibility and clinical outcomes of dupilumab injection interval extension in patients with CRSwNP followed for up to 60 months. The key findings indicate that extending the dosing interval beyond the standard Q2W regimen was feasible in the majority of selected clinically controlled patients. Specifically, 54% reached an interval of Q4W or longer (up to Q10W in isolated cases), and 23% who could not tolerate a Q4W interval were able to maintain a partially extended Q3W regimen. Both patient-reported quality of life (SNOT-22) and objective endoscopic findings (NPS) remained stable during follow-up, with no significant difference in longitudinal outcomes compared with patients who continued Q2W treatment.

These findings add important real-world data to the emerging evidence supporting the feasibility of dupilumab tapering in CRSwNP. De Corso et al. [23] reported that 48% of patients extended their dosing interval from Q2W to Q4W without losing clinical benefit. In the prospective Dutch cohort by van der Lans et al. [24], tapering was feasible in a large majority of patients, increasing from 79.5% at 24 weeks to 93.7% and 95.8% at 48 and 96 weeks, respectively; extension to Q6W–Q8W was achieved in 74% of patients, and a smaller subgroup even reached Q12W. Appel et al. [25] reported successful extension to Q4W in 29 patients, with nine of them subsequently reaching Q6W. Alghamdi et al. [26] showed that 39 of 42 patients maintained Q4W dosing for one year after prior stable Q2W treatment, while only three patients returned to Q2W. In a Korean study, Yoon et al. [27] found that an extension to at least Q4W was feasible in 91% of patients after 6 months, with 23% reaching intervals of at least Q8W. More recently, Campion et al. [28] reported long-term real-world outcomes over up to five years and found that 40% of patients extended their dosing interval without loss of efficacy. Our results are well in line with these reports, with 77% of patients maintaining an extended dosing interval beyond the standard Q2W regimen and 17% reaching intervals beyond Q4W up to Q10W.

The inclusion of patients maintained on continuous Q2W treatment provided additional context for interpreting our interval-extension outcomes. In both groups, SNOT-22 and NPS significantly improved after dupilumab initiation and remained stable throughout follow-up. Moreover, the longitudinal courses of both outcomes did not significantly differ between patients with an interval-extension attempt and those continuing Q2W treatment. These findings suggest that, in selected clinically stable patients, interval extension was not associated with a detectable loss of CRS-specific quality of life or nasal polyp control compared with continued standard dosing. However, this comparison should be interpreted with caution. As interval extension was offered at the treating physician’s discretion to clinically stable patients rather than allocated at random, the two groups may differ in unmeasured ways. Accordingly, the absence of a significant between-group difference indicates that no loss of disease control was detectable under interval extension, rather than proving equivalence or establishing formal non-inferiority, for which a randomized or prospective design would be required.

Several biological mechanisms may help explain why disease control in dupilumab therapy of CRSwNP can persist despite less frequent dosing. As previously suggested, this may be due to persistent IL-4Rα blockade by dupilumab, supported by relatively high serum concentrations after the initial biweekly treatment phase, high receptor availability, and the possibility that full IL-4Rα saturation is not always needed for clinical efficacy. In addition, the early reduction in polyp tissue and mucosal inflammation may lower the inflammatory burden, meaning that less drug exposure may be sufficient to maintain disease control. Patient-specific pharmacokinetic and pharmacodynamic differences may also explain why some patients tolerate longer intervals than others [24].

Identification of clinical predictors of successful interval extension is essential to guide patient selection in clinical practice. To our knowledge, apart from the recent study by Campion et al. [28], the present analysis is the only report to date that has systematically compared patients with and without successful interval extension to identify such predictors. In our cohort, no baseline characteristic differed significantly between the two groups, although some observations deserve mention. Coexisting N-ERD, baseline blood EOS and serum total IgE were higher among patients who did not tolerate interval extension to Q4W. These differences did not reach statistical significance, possibly due to the limited sample size, but they are biologically plausible and align with previous reports. Campion et al. [28] observed that comorbid N-ERD, a condition closely associated with severe asthma and a high type 2 inflammatory load, was the clinical factor most consistently linked to the need for dose re-intensification after initial extension. These observations suggest that patients with a higher cumulative type 2 inflammatory burden may require more frequent dosing to maintain control of the entire airway disease and should be monitored more closely if interval extension is attempted.

Several protocols for dupilumab interval extension in CRSwNP have been described in the existing literature. Two studies used a fixed de-escalation approach from the approved Q2W regimen to Q4W [23,26]. Huber et al. [29] applied an intermediate step by initially extending the dosing interval to Q3W and subsequently to Q4W, although the duration of the intermediate Q3W phase was not specified. Other studies applied more individualized strategies. Yoon et al. [27] used a symptom-guided strategy in which the standard dosing interval was extended in two-week steps from Q4W up to Q12W according to the SNOT-22 result at each monthly visit. In the PolyReg study, the injection interval was prolonged by two weeks at a time every six months in patients with a substantial treatment response, up to a maximum interval of Q12W [24]. In the present study, we used a comparable stepwise interval-extension protocol, increasing the dupilumab interval by two weeks every six months, provided that patients remained clinically stable and agreed to further prolongation. A unique and practical feature of our study was that some patients who did not tolerate Q4W independently transitioned on their own to a Q3W regimen, which was then maintained throughout follow-up. To our knowledge, this is the first study to describe a successful step-down to an intermediate dosing interval instead of a return to the prior regimen in patients in whom an attempted extension step was not tolerated. This observation suggests that intermediate intervals such as Q3W may represent a clinically valuable option in such situations, allowing patients to preserve part of the reduced treatment burden while still maintaining disease control. The same principle could be applied at higher extension levels, where intermediate steps, such as Q5W, Q7W, or Q9W, may be considered in future tapering protocols whenever a planned extension is not tolerated.

Criteria for initiating dupilumab interval extension remain insufficiently standardized. In clinical practice, most published strategies have considered tapering only after a relevant treatment response or stable disease control had been achieved. In the PolyReg study, tapering was initiated in patients with a moderate-to-excellent response according to EPOS 2020-based criteria [24], whereas De Corso et al. [23] and Huber et al. [29] based their extension strategies on the updated EPOS/EUOREA 2023 response criteria [21]. Yoon et al. [27] used a symptom-guided approach based on SNOT-22 scores, extending the interval when patients with a baseline SNOT-22 >40 achieved a score ≤20 or when those with a baseline SNOT-22 ≤40 achieved an improvement of at least 50%. Alghamdi et al. [26] initiated interval extension after clinical stability had been achieved, defined as a reduction in NPS by ≥1 point per side from baseline, without acute exacerbations or relevant symptom worsening needing SCS or ESS. In our cohort, interval extension was offered in patients with stable disease, according to the EPOS 2012 CRS control criteria [31]. In this context, the concept of CRS control may provide a useful framework for future tapering strategies [32]. A recent international Delphi study identified overall symptom severity, CRS-related systemic corticosteroid use, nasal obstruction severity, and patient-reported control as essential criteria for CRS control assessment [33]. In addition, the recently validated Chronic Rhinosinusitis Control Test may help standardize future control-based tapering decisions [34]. Future studies should evaluate whether such control-based criteria, alone or in combination with objective NPS status, or EPOS/EUFOREA response categories, can better identify patients most suitable for safe interval extension.

The optimal timing for initiating dupilumab interval extension is also not yet established. In the present real-world cohort, interval extension was introduced gradually as tapering strategies in CRSwNP emerged increasingly in the last years. Accordingly, patients had already received dupilumab at the standard dosage for different durations before interval extension became part of routine care, resulting in variable treatment durations ranging from 12 to 42 months. A similar pattern was seen in most of the published real-world studies, in which interval extension was started in the vast majority of cases after a minimum of 12 months treatment duration [24,25,26,28,29]. In contrast, Yoon et al. [27] adjusted dosing intervals already within the first months of treatment, which likely reflects the specific Korean healthcare context, where dupilumab for CRSwNP was not reimbursed and cost-effectiveness was therefore a major consideration. However, in our cohort, treatment duration before tapering initiation was not associated with successful interval extension. Similar findings were reported by Campion et al. [28]. De Corso et al. [23] further suggested that tapering based on good treatment response should preferably be considered only after at least 12 months of follow-up because sustained control cannot be reliably established earlier. This view is related to the emerging concept of remission in CRSwNP, which goes beyond short-term response and requires sustained symptom control and absence of active endoscopic disease over time. However, whether remission should serve as a prerequisite for tapering needs further investigation, as the prognostic value and clinical utility of remission in CRS have not yet been clearly established [35].

The rationale for considering dupilumab interval extension in routine clinical practice is based on several considerations. One important aspect is the economic burden of long-term treatment. In the Austrian healthcare context, a single 300 mg dose of dupilumab is currently priced at €556.38 [36]. Biweekly administration thus corresponds to approximately 26 injections a year and an annual cost of roughly €14,470 per patient. By comparison, Q3W lowers this annual cost to €9640, Q4W to €7230, Q6W to €4820, Q8W to €3620, and Q10W to about €2890 per patient and year. Over a five-year period, accounting for the stepwise progression to longer intervals, the cumulative saving across our 27 successfully extended patients reaches approximately €960,000. Once all patients have reached their respective maximum interval, the cohort’s annual saving approaches €194,000. Although these figures are simplified estimates and do not represent a formal pharmacoeconomic evaluation, they nevertheless illustrate the substantial financial impact that tapering may have over the long course of dupilumab therapy. Second, although dupilumab is generally considered safe and well tolerated, long-term treatment results in prolonged cumulative drug exposure. Even mild or uncommon adverse events may become more relevant when therapy is continued for many years. Reducing treatment frequency may therefore help lower cumulative exposure and the long-term burden of treatment-related adverse events without discontinuing an effective therapy. Third, many patients with CRSwNP belong to the working-age population, and the practical organization of repeated subcutaneous administrations, including cold-chain storage of the medication and the need to plan around professional, family, and travel obligations, can contribute to treatment fatigue and may reduce patients’ flexibility and quality of life. Interval extension may lower this burden and support long-term adherence.

Several limitations of the present study should be acknowledged. First, the retrospective single-center design and the relatively small sample size limit the generalizability of our findings and reduce the statistical power to identify predictors of extension success. Second, selection bias cannot be excluded, as tapering was offered only to clinically stable patients who may have had an inherently more favorable course. Third, although patients continuing Q2W treatment were included as a comparison group, this group does not represent a formal control group because treatment allocation was not randomized, interval extension was not systematically offered to all eligible patients, and documentation of whether interval extension had been offered or declined was incomplete. Therefore, the between-group comparison does not establish non-inferiority or comparative effectiveness. Fourth, the timing of interval extension and follow-up duration differed between patients, reflecting the real-world implementation of this strategy. As a result, fewer patients had long-term follow-up at later time points, and the longest intervals, such as Q8W and Q10W, were reached only in single individuals. These findings should therefore be interpreted with caution. Finally, clinically relevant outcomes, including symptom severity scores, olfactory testing, and asthma-specific outcomes, were not systematically assessed.

## 4. Materials and Methods

### 4.1. Study Design and Ethical Considerations

This retrospective, single-center, longitudinal cohort study was conducted at the Department of Otorhinolaryngology, Medical University of Graz, Austria. This study was conducted in accordance with the Declaration of Helsinki and approved by the Institutional Review Board of the Medical University of Graz. Patient consent was waived due to the retrospective nature of this study. Patient’s clinical records were anonymized prior to analysis.

### 4.2. Study Population

All adult patients with CRSwNP who received >12 months of dupilumab treatment (DUPIXENT^®^, SANOFI WINTHROP INDUSTRIE, 82 Avenue Raspail, 94250 Gentilly, France) at our department were retrospectively included in this study. CRSwNP was diagnosed according to the European Position Paper on Rhinosinusitis and Nasal Polyps (EPOS) [1].

### 4.3. Treatment Protocol

Dupilumab prescription followed the national reimbursement criteria set by the Austrian Federation of Social Insurances [36]. All study participants received at least 12 months of dupilumab treatment biweekly (Q2W). In clinically stable patients, interval extension was used as an individualized routine-care strategy at the discretion of the treating physician and in agreement with the patient. Clinical stability was defined according to the EPOS 2012 CRS control assessment concept, based on the absence of bothersome CRS symptoms and no requirement for rescue treatment, including systemic corticosteroids or salvage ESS [31]. Documentation was available for actual interval-extension attempts but not consistently for whether interval extension had been offered by the physician and/or declined by the patient. The injection interval was extended stepwise by two weeks (Q4W, Q6W Q8W, etc.), with at least six months between steps. Prior to each further extension, patients were asked whether they wished to proceed. Those who experienced severe symptom recurrence or clinical deterioration after a dose adjustment were instructed to independently self-return to their previously effective regimen (e.g., from Q4W back to Q2W). A subset of patients who could not tolerate Q4W independently transitioned to a Q3W regimen, which was retained throughout follow-up. As the practice of interval extension was implemented progressively at our center, the included patients started the extension regimen at varying stages of their overall treatment duration. The dupilumab dosing of 300 mg remained in all patients unchanged.

### 4.4. Follow-Up and Clinical Monitoring

Demographic and medical history data, including age, sex, prior ESS, and comorbidities, including asthma and NERD, were collected at baseline (defined as the biologic initiation visit). Patients were followed every 6 months (±6 weeks) for outcome monitoring. At baseline and at each follow-up visit, the Sino-Nasal Outcome Test-22 (SNOT-22) [37], Nasal Polyp Score (NPS) [38], blood eosinophil count (EOS), and serum total IgE level were assessed. SNOT-22 was used as a CRS-specific patient-reported outcome measure to assess health-related quality of life. It consists of 22 items scored from 0 to 5, resulting in a total score from 0 to 110, with higher scores indicating greater symptom burden [37]. Nasal polyp size was assessed using the Meltzer NPS, which grades polyp size from 0 to 4 on each side, resulting in a total score from 0 to 8; higher scores indicate greater nasal polyp burden [38].

### 4.5. Statistical Analyses

Statistical analyses were performed using SPSS software, version 29.0 (IBM Corp., Armonk, NY, USA) and R software 4.3.2 (R Foundation for Statistical Computing, Vienna, Austria). A two-sided *p*-value < 0.05 was considered statistically significant. Continuous variables were presented as means ± standard deviations or medians with interquartile ranges, as appropriate. Categorical variables were reported as absolute numbers and percentages. Longitudinal changes in SNOT-22 were analyzed using linear mixed-effects models with visit as fixed effect and patient ID as random intercept to account for repeated measurements within individuals. Pairwise comparisons with baseline were adjusted using Bonferroni correction. Longitudinal changes in NPS were analyzed using an ordinal mixed-effects model with cumulative logit link, visit as fixed effect, and patient ID as random intercept. The overall effect of visit was assessed using a likelihood-ratio test comparing the full model with a null model including only the random intercept. Pairwise comparisons against baseline were performed using likelihood-ratio tests and adjusted using Bonferroni correction. To assess clinical outcome during interval extension, SNOT-22 and NPS values were compared at three predefined time points: baseline, pre-extension, and post-extension. For SNOT-22, repeated-measures ANOVA was used; Greenhouse–Geisser correction was applied when the assumption of sphericity was violated, and pairwise comparisons were adjusted using Bonferroni correction. For NPS, the Friedman test was used, followed by Bonferroni-corrected pairwise comparisons. For comparisons between patients who reached ≥Q4W and those who did not tolerate Q4W, independent *t*-tests or Welch’s *t*-tests were used for continuous variables, depending on variance equality. Categorical variables were compared using Pearson’s chi-squared test or Fisher’s exact test when expected cell frequencies were below 5. In an additional analysis, patients were stratified into unsuccessful extension (Q2W), partial extension (Q3W after inability to tolerate Q4W), and ≥Q4W. Group comparisons were performed using one-way ANOVA or Kruskal–Wallis tests for continuous variables and chi-squared or Fisher’s exact tests for categorical variables, as appropriate. For the comparison between patients with an interval-extension attempt and those continuing Q2W treatment without an attempt, combined mixed-effects models were calculated for SNOT-22 and NPS. Group, visit, and the group-by-visit interaction were included as fixed effects, with patient ID as random intercept. The group-by-visit interaction was used to assess whether the longitudinal outcome trajectories differed between groups.

## 5. Conclusions

In this real-world study, dupilumab interval extension was feasible in the majority of selected, clinically controlled patients with CRSwNP. A maintained interval of Q4W or longer was achieved in 54% of patients (up to Q10W in isolated cases), and a further 23% who could not tolerate Q4W remained on a partially extended Q3W regimen. Throughout follow-up, CRS-specific quality of life and nasal polyp burden remained stable, no patient required rescue treatment, and outcomes did not differ significantly from those of patients who continued standard Q2W dosing. A notable observation is that patients who did not tolerate a planned extension step were able to maintain disease control on intermediate intervals such as Q3W, suggesting that smaller individualized adjustments may be a useful option in such situations. Overall, our findings support dupilumab interval extension as a potential long-term management strategy that may reduce treatment burden, cumulative drug exposure, and healthcare costs without compromising disease control. Larger prospective and controlled studies are needed to standardize tapering criteria, define the optimal protocol for extension and identify reliable predictors of successful interval prolongation.

## Figures and Tables

**Table 1:** Demographics and baseline clinical characteristics.

Characteristic	Interval-Extension Attempt n = 35	No Interval-Extension Attempt n = 30	*p*-Value
Sex, w/m	10 (29%)/25 (71%)	16 (53%)/14 (47%)	0.075
Age at start of treatment (years)	54.6 ± 13.3	49.1 ± 15.4	0.120
n° previous ESS (0/1/2/3/4/5/6)	1 (3%)/13 (37%)/8 (23%)/6 (17%)/4 (11%)/3 (9%)	1 (3%)/12 (40%)/7 (23%)/6 (20%)/1 (3%)/2 (7%)/1 (3%)	0.915
Blood EOS (×10^9^/L)	0.40 (0.25–0.65)	0.45 (0.35–0.60)	0.380
Blood EOS ≥ 0.15 × 10^9^/L	32 (91%)	27 (90%)	1.000
Serum total IgE (kU/L)	114.0 (79–224)	149 (80–288)	0.382
Serum total IgE ≥ 100 kU/L	21 (60%)	19 (63%)	0.783
Coexisting asthma	17 (49%)	25 (83%)	0.005
Coexisting N-ERD	3 (9%)	6 (20%)	0.282
SNOT-22	48.5 ± 20.0	57.9 ± 21.9	0.075
NPS	5 (4–6)	5 (4–6)	0.954

Continuous parameters are presented as means ± standard deviations or median plus interquartile range (IQR). Categorical parameters are presented as absolute numbers and percentages (%). w: women. m: men. ESS: endoscopic sinus surgery. EOS: eosinophil count. N-ERD: NSAID-exacerbated respiratory disease. SNOT-22: Sino-Nasal Outcome Test-22. NPS: Nasal Polyp Score.

**Table 2:** Longitudinal SNOT-22 outcomes in patients with an interval-extension attempt.

Visit	n	Mean SNOT-22	*p*-Value vs. Baseline
Baseline	35	48.4 ± 20.0	reference
6 months	35	25.0 ± 18.2	<0.001
12 months	35	18.8 ± 13.1	<0.001
18 months	35	17.8 ± 13.8	<0.001
24 months	27	14.9 ± 14.1	<0.001
30 months	20	16.7 ± 11.8	<0.001
36 months	12	10.5 ± 10.5	<0.001
42 months	11	8.6 ± 10.1	<0.001
48 months	7	15.1 ± 13.6	<0.001
54 months	6	14.3 ± 13.7	<0.001
60 months	3	8.3 ± 2.5	<0.001

SNOT-22 values are presented as means ± standard deviation. *p*-values were derived from the mixed-effects model and refer to comparisons with baseline after Bonferroni correction.

**Table 3:** Longitudinal NPS outcomes in patients with an interval-extension attempt.

Visit	n	Median NPS (IQR)	*p*-Value vs. Baseline
Baseline	35	5 (4–6)	reference
6 months	35	1 (0–2)	<0.001
12 months	35	0 (0–2)	<0.001
18 months	35	0 (0–2)	<0.001
24 months	27	0 (0)	<0.001
30 months	20	0 (0)	<0.001
36 months	12	0 (0)	<0.001
42 months	11	0 (0–1)	<0.001
48 months	7	0 (0)	0.003
54 months	6	0 (0)	0.010
60 months	3	0 (0)	0.039

NPS values are presented as medians with interquartile ranges (IQR). *p*-values were derived from the mixed-effects model and refer to comparisons with baseline after Bonferroni correction.

**Table 4:** Dupilumab interval-extension results.

Interval-Extension Outcome	n (%)
**Extension to Q4W or longer**	**19 (54%)**
Q4W	13 (37%)
Q6W	4 (11%)
Q8W	1 (3%)
Q10W	1 (3%)
**Partial extension Q3W** (after inability to tolerate Q4W)	8 (23%)
**Unsuccessful extension (return to Q2W)**	**8 (23%)**

**Table 5:** Comparison of clinical characteristics across interval-extension groups.

Parameter	≥Q4W n = 19	Q3W n = 8	Return to Q2W n = 8	<Q4W n = 16	*p*-Value *
Sex, w/m	6 (32%)/13 (68%)	1 (13%)/7 (87%)	3 (37%)/5 (63%)	4 (25%)/12 (75%)	0.668
Age at start of treatment (years)	55.8 ± 13.8	54.0 ± 11.8	53.2 ± 14.7	53.6 ± 12.9	0.669
n° previous ESS (0/1/2/3/4/5)	1 (5%)/7 (37%)/3 (16%)/5 (26%)/1 (5%)/2 (11%)	0 (0%)/3 (37.5%)/3 (37.5%)/1 (12.5%)/1 (12.5%)/0 (0%)	0 (0%)/3 (37%)/2 (25%)/0 (0%)/2 (25%)/1 (13%)	0 (0%)/6 (37%)/5 (31%)/1 (6%)/3 (19%)/1 (6%)	0.741
Baseline blood EOS (×10^9^/L)	0.3 (0.2–0.4)	0.4 (0.2–0.8)	0.5 (0.2–0.9)	0.4 (0.2–0.9)	0.398
Baseline blood EOS ≥0.15 ×10^9^/L	18 (94%)	6 (75%)	8 (100%)	14 (87%)	0.582
Baseline total IgE (kU/L)	97.7 (60–214)	135 (76–188)	233.5 (110–408)	135 (96–289)	0.623
Serum total IgE ≥100 kU/L	9 (47%)	5 (62%)	7 (87%)	12 (75%)	0.166
Coexisting asthma	9 (47%)	2 (25%)	6 (75%)	8 (50%)	0.877
Coexisting N-ERD	0 (0%)	2 (25%)	1 (12%)	3 (19%)	0.086
Baseline SNOT-22	43.8 ± 17.3	52.6 ± 22.5	55.3 ± 23.1	54.0 ± 22.0	0.147
Baseline NPS	5 (4–6)	6 (4–8)	4 (4–5.5)	4.5 (4–6)	0.315
Treatment duration to first interval extension attempt, months	18 (12–24)	15 (12–24)	18 (15–19)	18 (12–22)	0.865

Continuous parameters are presented as means ± standard deviations or median plus interquartile range (IQR). Categorical parameters are presented as absolute numbers and percentages (%). w: women. m: men. ESS: endoscopic sinus surgery. EOS: eosinophil count. N-ERD: NSAID-exacerbated respiratory disease. SNOT-22: Sino-Nasal Outcome Test-22. NPS: Nasal Polyp Score. * *p*-values refer to the comparison ≥Q4W vs. <Q4W.

## Data Availability

The raw data supporting the conclusions of this article will be made available by the authors on reasonable request.

## Supplementary Materials

The following supporting information can be downloaded at: https://www.mdpi.com/article/10.3390/ph19060961/s1, Table S1: Longitudinal SNOT-22 outcomes in patients without an interval-extension attempt; Table S2: Longitudinal NPS outcomes in patients without an interval-extension attempt.
