# Supplementary material for: Extension of Dupilumab Injection Intervals in Chronic Rhinosinusitis with Nasal Polyps: A Real-World Study
Source: Pharmaceuticals (Basel). 2026 Jun 22;19(6):961. doi: 10.3390/ph19060961 (PMC13305528; doi:10.3390/ph19060961)
Supplement: Supplementary file 1 [file pharmaceuticals-19-00961-s001.zip › pharmaceuticals-4339358-supplementary.pdf]

Supplementary Material

Table S1. Longitudinal SNOT-22 outcomes in patients without an interval-extension attempt

Visit	n	Mean SNOT-22	p value vs baseline
Baseline	30	57.9 ± 21.9	reference
6 months	30	24.7 ± 19.8	<0.001
12 months	30	21.9 ± 16.7	<0.001
18 months	19	18.2 ± 11.2	<0.001
24 months	10	16.5 ± 14.9	<0.001
30 months	8	17.3 ± 17.9	<0.001
36 months	4	14.2 ± 11.1	<0.001
42 months	2	11.5 ± 13.4	<0.001
48 months	2	12.5 ± 16.2	<0.001
54 months	1	18	
60 months	0	-	-

SNOT-22 values are presented as means ± standard deviation. p-values were derived from the mixed-effects model and refer to comparisons with baseline after Bonferroni correction.

Table S2. Longitudinal NPS outcomes in patients without an interval-extension attempt

Visit	n	Median NPS (IQR)	p value vs baseline
Baseline	30	5 (4-6)	reference
6 months	30	1 (0-2)	<0.001
12 months	30	0.5 (0-1)	<0.001
18 months	19	0 (0-1)	<0.001
24 months	10	0 (0-1)	<0.001
30 months	8	0 (0-1)	<0.001
36 months	4	0 (0-1)	<0.001
42 months	2	0 (0)	<0.001
48 months	2	0.5 (0-1)	<0.001
54 months	1	0	
60 months	0	-	

NPS values are presented as medians with interquartile ranges (IQR). p-values were derived from the mixed-effects model and refer to comparisons with baseline after Bonferroni correction.
